# OR11-001 - Protein misfolding in mevalonate kinase deficiency

**DOI:** 10.1186/1546-0096-11-S1-A190

**Published:** 2013-11-08

**Authors:** S Stojanov, SW Gersting, L Reitzle, D Reiß, BH Belohradsky, M Vogeser, B Maier, D Teupser, AC Muntau

**Affiliations:** 1 Department of Molecular Pediatrics, Dr. von Hauner Children's Hospital, Ludwig-Maximilians-University, Munich, Germany; 2 Department of Infectious Diseases and Immunology, Dr. von Hauner Children's Hospital, Ludwig-Maximilians-University, Germany; 3 Institute of Laboratory Medicine, Ludwig-Maximilians-University, Munich, Germany

## Introduction

Mevalonate kinase deficiency (MKD) is an early-onset autosomal recessive autoinflammatory fever syndrome lacking specific treatment options. Current data point to protein misfolding as underlying molecular mechanism.

## Objectives

To characterize the molecular pathophysiology of eight MK variants associated with a hyperimmunoglobulinemia D with periodic fever syndrome (HIDS) (W188X, V203A, V377I, H380R) and/or mevalonic aciduria (MA) (H20P, L264F, I268T, A334T) phenotype.

## Methods

Recombinant wild-type (WT) and variant MK proteins N-terminally fused to a maltose binding protein (MBP) were expressed in *E. coli*. Following affinity purification and size exclusion chromatography, the MBP-MK fusion (fMK) or cleaved MK (cMK) proteins, respectively, were characterized with regard to oligomerization, thermal unfolding monitored by differential scanning fluorimetry, thermal aggregation analyzed by right angle light scattering, and enzyme activity. These data were correlated with the disease phenotype and MK activities measured in blood cells and/or fibroblasts of 101 homozygous or compound heterozygous MKD patients, respectively, that were collected retrospectively from our center and the literature.

## Results

MK dimer assembly was impaired in all analyzed MK variants. Oligomerization profiles of V377I and V203A were most similar to that of WT, A334T showed reduced amounts of fMK and cMK dimers, while H20P and I268T revealed only small peaks of fMK dimers, and W188X, H380R, and L264F lacked any dimers, thus pointing to altered protein conformation of various degree. This was confirmed by partial unfolding in the native state, and variably accelerated thermal unfolding of all MK variants. Furthermore, thermal aggregation kinetics investigated in cMK V203A, A334T, and V377I, respectively, were altered. Catalytic function varied from high residual activity in cMK V377I (100% of WT), to moderately decreased activity in cMK V203A (70%) and fMK I268T (80%), markedly decreased activity in cMK A334T (3.1%), and almost no activity in fMK H20P (0.4%), as well as W188X (0.7%), L264F (0.9%), and H380R (1.5%). Consistently, MKD patients carrying at least one V377I mutation presented the HIDS phenotype, while those being compound heterozygous or homozygous for H20P, L264F, I268T, and/or A334T mutations were associated with MA. While patient MK activities were highest among V377I homozygous or V377I/I268T and V377I/H20P genotypes, there was a considerable variability of MK activities for other genotypes analyzed being associated with HIDS or MA.

## Conclusion

These results support the hypothesis of protein misfolding with loss of function being the molecular basis in MKD, and thus may assist the development of novel targeted therapeutic strategies.

## Competing interests

None declared.

